# Trypanophobia: Traumatic spondylolisthesis following phlebotomy - A case report

**DOI:** 10.51866/cr.313

**Published:** 2024-09-18

**Authors:** Chitra Suluraju, Alidahani Mohamad Yusof

**Affiliations:** 1 MD, MMed (Family Medicine), Klinik Kesihatan Petaling Bahagia, Jalan Puchong, Kuala Lumpur, Wilayah Persekutuan Kuala Lumpur, Malaysia.; 2 MBBS, MMed (Family Medicine), Klinik Kesihatan Petaling Bahagia, Jalan Puchong, Kuala Lumpur, Wilayah Persekutuan Kuala Lumpur, Malaysia.

**Keywords:** Trypanophobia, Hangman's Fracture, Vasovagal Syncope, Phlebotomy, Needle Phobia

## Abstract

Injuries to the cervical spine following phlebotomy have not been reported in the literature. Herein, we report the case of a 27-year-old lady with major depressive disorder and trypanophobia who experienced a syncopal episode during a blood test, resulting in a C2 Hangman’s fracture. After the application of a halo vest, she recovered without any long-term pain or neurological impairments. The concept of needle phobia is important for healthcare professionals to understand, since patients’ fear of needles can lead to detrimental injuries during panic attacks. Greater attention should be paid to alleviate this fear, with the ultimate goals of improving health and preventing unwanted injuries following phlebotomy.

## Introduction

Traumatic spondylolisthesis of the axis, commonly referred to as a Hangman’s fracture, occurs due to a bilateral fracture of the C2 pars interarticularis. This injury constitutes 4%–7% of all cervical spinal fractures and 20%-22% of all axis fractures.^1-3^ In 1965, Schneider coined the term “Hangman’s fracture” to describe this fracture pattern due to similarities with fractures associated with judicial hangings^4^; however, it has been found that this fracture pattern is seen only in about 10% of injuries associated with hangings.^5^ The fracture can be significantly unstable. Without treatment, the bone may shift, leading to deformities that result in serious damage to the spinal cord or progressive pain.

The fear of needles, also known as trypanophobia, is an extreme fear of medical procedures involving needles. A previous global survey of the general adult population revealed that 63.2% (n=1325) reported experiencing needle phobia, with the majority stating avoidance of blood draws (52.2%), blood donations (49.0%) and vaccinations (33.1%).^6^ Consequently, there is growing concern within the healthcare system about this condition, leading to its recognition and inclusion under the diagnostic category of blood-injection-injury phobia in the American Psychiatric Association’s Diagnostic and Statistical Manual of Mental Disorders, Fourth Edition in 1994.^7^ The most common cause of this phobia is an inherited vasovagal reflex triggered by needle piercing. This inherited reflex becomes evident after several exposures to needles.

Hence, the reaction is both an inherited and a learnt behaviour.^8^ Compared to other phobias, wherein patients experience tachycardia following exposure to the feared object, needle phobia typically involves anticipatory tachycardia and hypertension for a short period before needle insertion. These are often followed by bradycardia and hypotension on needle insertion and may be accompanied by pallor, tinnitus, diaphoresis, syncope and, in severe cases, even systole or death.^9^ Injuries to the cervical spine associated with phlebotomy have not been described in the literature.

Herein, we present a case of a Hangman’s fracture resulting from an injury due to a vasovagal attack triggered by trypanophobia in a 27-year-old woman.

## Case presentation

A 27-year-old lady with underlying major depressive disorder came in for a blood test on the day of the incident. She had trypanophobia, which contributed to a vasovagal attack she experienced following COVID-19 vaccination within the past year. Otherwise, she had no risk factors for osteoporosis or history of neck injury. On the day of the procedure, she was advised to relax and lie her head down on the table to calm herself during the blood draw.

Unfortunately, the patient experienced a syncopal episode during phlebotomy. She fell forward with her neck hyperextended and landed on the floor in a prone position. After the fall, she complained of pain in the back of her neck but did not experience any weakness or numbness. She could ambulate without assistance following the fall. No chest pain, dyspnoea, giddiness, blurring of vision, headache or vomiting was noted.

On examination, the patient was alert, conscious and co-operative. Her blood pressure was 109/66 mmHg; pulse rate, 96 beats per minute; respiratory rate, 18 breaths per minute; and SPO2 level, 100% on room air. Her capillary blood sugar level was 7.6 mmol/L. Her bilateral pupils were sized 3 mm and reactive. There was mild local tenderness over the C2 level. Otherwise, no external skin changes or bruises were noted. There were also no other injuries or neurological deficits observed. Her gait was normal, and her respiratory and cardiovascular examinations were unremarkable.

A cervical collar was applied due to suspicion of a cervical injury, and she was referred to Kuala Lumpur General Hospital for further evaluation and observation. The orthopaedic team assessed her and found no neurological deficit. Her muscle strength was categorised as grade V on the Medical Research Council Scale for Muscle Strength, and her sensation was normal. Radiographic imaging showed a C2 Hangman’s fracture. Subsequently, cervical computed tomography was conducted, which confirmed the C2 Hangman’s fracture with involvement of the foramen transversarium and further revealed a fracture of the bilateral C2 interarticularis extending into the left transverse process and left foramen transversarium.

The patient was admitted to the ward for pain management and immobilisation and was scheduled for a halo vest application under sedation. She was also referred to the medical team for a second opinion regarding her syncopal episode. The medical team diagnosed it as a vasovagal attack secondary to trypanophobia and subsequently discharged her. The patient wore a halo vest for 3 months and recovered well without any neurological deficit or chronic pain. She successfully discontinued her antidepressants and continued to see her counsellor for follow-up. She also learnt relaxation techniques. Healthcare staff were educated about needle phobia and ways to reduce such fear and trauma, such as using smaller needles, positioning patients on a couch or blood collection chair during blood draws, allowing ample time for observation, providing distractions and explaining procedures.

## Discussion

The estimated prevalence of needle fear ranges from 20% to 50% in adolescents and from 20% to 30% in young adults. In general, the prevalence decreases with increasing age. Both needle fear and needle phobia are more prevalent in female populations than in male populations.^10^

According to the Diagnostic and Statistical Manual of Mental Disorders, Fifth Edition, Text Revision, there are several reasons why people might develop trypanophobia.^11^ The possible causes include experiencing a traumatic event involving needles, observing a traumatic event involving needles, experiencing an unexpected panic attack during a medical procedure involving needles or learning too much information about negative events involving needles. Additionally, genetic factors may play a role in the development of such phobia.^11^

Individuals with significant anxiety may experience a vasovagal reaction to situations involving needles. This reaction occurs when individuals faint after needle exposure due to their heart rate and blood pressure rapidly increasing before dropping abruptly.

According to the Centers for Disease Control and Prevention, patient preparation and support as well as pain management can help address milder cases of needle phobia. These involve educating patients about the procedure and the role of needles; explaining what will happen before, during and after the procedure; identifying the causes of their fear; avoiding potential triggers; and managing pain using numbing creams, vibration, distraction or relaxation techniques.^12^ For example, if a person with a fear of needles needs to undergo a procedure involving one, a healthcare professional may offer them a beverage, a snack or some reassurance about the procedure to help prevent fainting. Additionally, the healthcare professional should advise the person to sit on a chair or lie down and remain under close supervision after the procedure.

The present case of the 27-year-old woman with major depressive disorder and trypanophobia, who experienced a syncopal episode during a blood test resulting in a C2 Hangman’s fracture, underscores the significant impact of needle fear on patient safety and well-being. Existing literature suggests that needle fear, which is especially prevalent among female individuals, can stem from various factors including traumatic experiences and genetic predispositions. Vasovagal reactions, such as fainting, can occur in individuals with needle phobia. Effective management strategies include patient education, preparation and support as well as pain management techniques such as the usage of numbing creams and relaxation methods. Healthcare providers play a crucial role in addressing needle phobia by providing reassurance, minimising triggers and ensuring appropriate post-procedural care to prevent adverse events such as syncope.

## Conclusion

Due to trypanophobia being associated with physical complications, venous puncture or injection among affected patients becomes a complex procedure that should be performed by trained personnel. This is to ensure that the procedure is performed safely and competently to minimise trauma. Trypanophobia may cause individuals to avoid healthcare procedures such as vaccinations and blood tests. To help overcome this aversion, such individuals can undergo exposure therapy, which involves gradually increasing their exposure to needles in a safe environment.

## References

[1] Ge C, Hao D, He B (2015). Anterior cervical discectomy and fusion versus posterior fixation and fusion of C2-3 for unstable Hangman’s fracture.. J Spinal Disord Tech..

[2] Gehweiler JA, Clark WM, Schaaf RE (1979). Cervical spine trauma: the common combined conditions.. Radiology..

[3] Al-Mahfoudh R, Beagrie C, Woolley E (2015). Management of typical and atypical hangman’s fractures.. Glob Spine J..

[4] Schneider RC, Livingston KE, Cave AJ (1965). “Hangman’s fracture” of the cervical spine.. J Neurosurg..

[5] James R, Nasmyth-Jones R (1992). The occurrence of cervical fractures in victims of judicial hanging.. Forensic Sci Int..

[6] Alsbrooks K, Hoerauf K (2022). Prevalence, causes, impacts, and management of needle phobia: an international survey of a general adult population.. PLoS One.

[7] American Psychiatric Association. (1994). Diagnostic and Statistical Manual of Mental Disorders: DSM-IV..

[8] Hamilton JG (1995). Needle phobia: a neglected diagnosis.. J Fam Pract..

[9] Ellinwood EH, Hamilton JG (1991). Case report of a needle phobia.. J Fam Pract..

[10] McLenon J, Rogers MAM (2019). The fear of needles: a systematic review and meta-analysis.. J Adv Nurs..

[11] Diagnostic and Statistical Manual of Mental Disorders, 5 th Edition, Text Revision (DSM-5-TR).

[12] Centers for Disease Control and Prevention. (2023). Needle Fears and Phobia..

